# Personality, opinion strength, and social media use – not such a straightforward relationship

**DOI:** 10.1080/00049530.2025.2451156

**Published:** 2025-01-20

**Authors:** Melissa Cox, Bernadine Cocks, Susan E. Watt, Elizabeth C. Temple

**Affiliations:** School of Psychology, University of New England, Armidale, Australia

**Keywords:** Social media, personality, opinion strength, opinion direction, progressive opinions, big five, Dark Triad

## Abstract

**Objective:**

This study investigated the link between personality, opinions on social and political issues, and social media use, as well as the moderating effects of social media use on the relationship between personality and those opinions. Past research suggests that personality, opinion direction (i.e. favourability of an issue), and social media use are inter-related. However, the relationship between personality and opinion strength (i.e. how extreme an opinion is disregarding favourability), and potential moderating effects of social media use on that relationship have yet to be investigated.

**Method:**

Participants (N = 536) completed surveys measuring social media usage, personality, and opinions on various social issues.

**Results:**

Several personality traits predicted opinion direction or strength on at least one social issue. When all social issues were combined to measure overall progressive opinions, openness and extraversion predicted opinion direction, and openness predicted opinion strength. Time spent on social media significantly predicted direction of opinions on several issues, as well as strength of opinion on the issue of gender equality, however it did not moderate any relationship between personality and opinion direction or strength.

**Conclusions:**

Although opinions, personality, and social media use are sometimes related, individuals high or low in particular personality traits are at no greater risk of polarising due to social media use than anyone else.

## KEYPOINTS

**What is already known about this topic:**

1. Opinions on socially relevant topics have been found to relate to levels of particular personality traits, most commonly openness.
2. Several personality traits are related to social media use.
3. Polarisation of opinion can occur on social media.

**What this topic adds:**

1. The relationships previously identified in the literature are also present within an Australian sample.
2. Opinion strength, rather than just opinion direction, can be predicted by certain personality traits.
3. Social media use does not moderate the relationship between personality and opinion.

Opinions can be a determining factor in behaviour (e.g., see the Theory of Planned behaviour; Ajzen & Schmidt, 2020). When those opinions are extreme, opinion-congruent behaviour is more likely to follow. If these opinions are prosocial, or one of positive personal consequence, that behaviour can be positive – for example, smokers with stronger attitudes towards quitting are more likely to quit smoking than those with weaker attitudes (Rahmawati et al., 2022). However, extreme opinions and beliefs have also been associated with heinous acts, such as the hate crimes perpetrated against Asian Americans during the COVID-19 pandemic (Han et al., 2023) and, more recently, the reported ongoing hate crimes against Palestinian and Jewish people (Cineas, 2023; Kassam, 2023). Prior research has linked both social media use (e.g., Dylko et al., 2018; Jiang, 2022; Quattrociocchi et al., 2016) and personality (e.g., Correa et al., 2010; Gil de Zúñiga et al., 2017) to the formation and maintenance of strong opinions. Personality is also associated with social media use (e.g., Gil de Zúñiga et al., 2017; Roos, 2023). However, it is unclear how personality, social media use, and strong opinions interrelate. Investigation of these associations is important to increase understanding of the factors contributing to the development of extreme opinions, to both increase the effectiveness of pro-social (e.g., health) information disseminated by social media and reduce the perpetration of hate crimes and other heinous acts related to social media use.

## Personality and opinions

The Big Five personality traits (i.e., extraversion, neuroticism, openness, conscientiousness, & agreeableness) are ubiquitous in personality research, as they account for as much variability in personality as possible in as few factors as possible (Soto & Jackson, 2020). More recently, the Dark Triad (i.e., Machiavellianism, narcissism, & psychopathy) have become a focus of personality research, with the studies published on the topic increasing year on year since the turn of the century (Muris et al., 2017). Most past research investigating associations between personality and opinions has focused on issues of societal or political importance. To identify such issues for the present study’s Australian sample, we utilised the ABC Vote Compass, which is a tool that allows Australian voters to understand how their opinions on key issues align with the major political parties (Australian Broadcast Corporation [ABC], 2019). The specific topics chosen for investigation were immigration, climate change, gender equality, and religious freedoms.

Such past research has examined relationships between personality traits, including both the Big Five and the Dark Triad on some opinion types (e.g., climate change, immigration) while other topics have yet to be examined (e.g., gender equality and religious freedoms). As can be seen in Table 1, for example, previous research has identified that openness, extraversion, and agreeableness often correlate positively with progressive opinions on climate change and immigration, while Machiavellianism, narcissism and psychopathy typically correlate negatively. Other traits, such as conscientiousness and neuroticism, relate to opinions on some topics but not others. Some of these relationships are, however, quite weak, thus further exploration is needed to better understand how personality and opinion interact.

**Table 1.** Brief summary of some of the research relating personality traits to opinions on climate change and immigration.

Trait		Pro-environmental/climate change	Immigration	
	*r*	Study	*r*	Study
Extraversion	.09 *ns*	Soutter et al. (2020) Annalakshmi et al. (2020)^a^	.33 *ns*	Stürmer et al. (2013) Hodson et al. (2009)
Openness	.22 .19	Soutter et al. (2020) Annalakshmi et al. (2020)^a^	.30 −.18	Stürmer et al. (2013) Hodson et al. (2009)
Agreeableness	.15 *ns*	Soutter et al. (2020) Annalakshmi et al. (2020)^a^	*ns* −.13	Stürmer et al. (2013) Hodson et al. (2009)
Conscientiousness	.12 *ns*	Soutter et al. (2020) Annalakshmi et al. (2020)^a^	*ns* *ns*	Stürmer et al. (2013) Hodson et al. (2009)
Neuroticism/emotionality	*ns* *ns*	Soutter et al. (2020) Annalakshmi et al. (2020)^a^	−.14^*b*^ *ns*	Stürmer et al. (2013) Hodson et al. (2009)
Psychopathy	−.23 −.13	Huang et al. (2019) Arvan (2013)	.20 *ns*	Hodson et al. (2009) Arvan (2013)
Narcissism	−.12 .07	Huang et al. (2019) Arvan (2013)	.19 .15	Hodson et al. (2009) Arvan (2013)
Machiavellianism	−.23 .09	Huang et al. (2019) Arvan (2013)	.19 .17	Hodson et al. (2009) Arvan (2013)

Stürmer et al. (2013) investigated xenophilia, or pro-immigration attitudes, while Hodson et al. (2009) investigated prejudice against immigrants, and Arvan (2013) investigated anti-immigration attitudes, explaining the different directions in the relationships. All relationships reported were significant at *p* < .05. Non-significant findings are denoted by *ns*..

^*a*^Calculated from the provided *t* statistic.

^*b*^Correlation coefficient relates to emotionality in the HEXACO model, rather than neuroticism in the Big Five model. Emotionality is strongly negatively correlated to neuroticism, so it can be inferred that a negative correlation between pro-immigration attitudes and emotionality suggests a positive correlation between pro-immigration attitudes and neuroticism.

Past research on opinions largely focuses on the direction of an individual’s opinion (e.g., opinions measured on a Likert scale ranging from *unfavourable* to *favourable*, see Annalakshmi et al., 2020; Hodson et al., 2009; Stürmer et al., 2013), rather than the strength of the opinion, regardless of direction. This provides a new avenue for investigation and exploration, as understanding whether particular personality traits increase the likelihood of forming strong opinions – regardless of direction – would provide insight into who is more or less likely to polarise (i.e., take a more extreme stance) on particular issues. As extreme opinions resulting from polarisation can relate to extreme actions such as racially based hate crimes, such a relationship is important to understand.

## Personality and social media

Investigations into the relationship between personality and the use of social media have also yielded inconsistent results. Individuals higher in extraversion, openness, neuroticism, and narcissism tend to use social media more than those that are low in those traits; however, there is a lack of consensus relating to agreeableness and conscientiousness (see Table 2 for specific research and effect sizes). The inconsistent results relating to agreeableness and conscientiousness may relate to the different ways each study measures general, that is, not problematic or addictive, social media use (e.g., global use, see Liu & Campbell, 2017; intensity of use, see; Lin et al., 2017; frequency of use, see Roos, 2023). However, the consistent findings relating to extraversion, openness, neuroticism, and narcissism suggest that regardless of how social media is used, people higher in these traits are likely to use it more.

**Table 2.** Summary of recent research into the relationship between personality and social media use.

Trait	Relationship	Source
Extraversion	*r* = .11 *r* = .14 *r* = .12 β = .076	Liu and Campbell (2017)* Lin et al. (2017) Roos (2023) Gil de Zúñiga et al. (2017)
Openness	*r* = *ns* *r* = .21 *r* = .13 β = .056	Liu and Campbell (2017)* Lin et al. (2017) Roos (2023) Gil de Zúñiga et al. (2017)
Neuroticism	*r* = .09 *r* = .08 *r* = .07 β = −.097	Liu and Campbell (2017)* Lin et al. (2017) Roos (2023) Gil de Zúñiga et al. (2017)^#^
Conscientiousness	*r* = *ns* *r* = *ns* *r* = −.16 β = .036	Liu and Campbell (2017)* Lin et al. (2017) Roos (2023) Gil de Zúñiga et al. (2017)
Agreeableness	*r* = *ns* *r* = −.09 *r* = −.09 β = .116	Liu and Campbell (2017)* Lin et al. (2017) Roos (2023) Gil de Zúñiga et al. (2017)
Narcissism	*r* = .074 *r* = .203 *r* = .11 *r* = .196 *r* = *ns* *r* = .19 *r* = .11	Mas Manchón and Davila (2022) – NPQC-R Mas Manchón and Davila (2022) – NPI-13 McCain and Campbell (2018)^^^ – meta-analysis (grandiose narcissism) Martingano et al. (2022) Chung et al. (2019) Fox and Rooney (2015) Servidio et al. (2021)
Machiavellianism	*r* = *ns* *r* = .13 *r* = *ns*	Chung et al. (2019) Fox and Rooney (2015) Servidio et al. (2021)
Psychopathy	*r* = *ns* *r* = .09 *r* = *ns*	Chung et al. (2019) Fox and Rooney (2015) Servidio et al. (2021)

Research exclusively examining problematic social media use, internet addiction, and similar pathological levels of social media use was excluded, as they are different constructs to the type of social media use included in this research. Only research relating to general social media use (e.g., duration, frequency, etc) was included. All relationships reported were significant at *p* < .05. Non-significant findings are denoted by *ns*..

*Liu and Campbell (2017) conducted a meta-analysis on several different measures of social media use. The statistics stated in this table relate to “global use”.

^#^This study measured emotional stability, which is strongly negatively correlated with neuroticism. This explains the different direction in the relationship.

^^^McCain and Campbell (2018) conducted a meta-analysis, and this statistic refers specifically to their findings around grandiose narcissism.

## The role of social media in opinion polarisation

Social media use has been shown to encourage opinion polarisation under the right circumstances, typically when users join *echo chambers* consisting of only users who hold similar opinions about an issue (Quattrociocchi et al., 2016; Schmidt et al., 2018). Many social media users deliberately seek out sources of information that agree with their viewpoints (Chong & Druckman, 2010), which can exacerbate this effect, as repeated exposure to opinion-congruent information can result in stronger opinions (Dylko et al., 2018). This opinion polarisation has been shown to occur for both general political opinions (Hong & Kim, 2016; Kubin & von Sikorski, 2021) and climate change (Williams et al., 2015). While this may be desirable for some circumstances such as reducing smoking, polarisation resulting in harm (e.g., inciting hatred and violence) can have extremely detrimental effects on both individuals and larger groups. The Christchurch massacre, for example, has been at least partly attributed to polarisation related to social media use (Royal Commission of Inquiry into the Attack on Christchurch Mosques, 2019). It is thus clear that better understanding of how social media impacts opinion is imperative, to both improve pro-social behaviours and reduce anti-social behaviours.

## The current research

Although past research into social media use, personality and opinion has yielded contradictory results, it appears likely that this is related to methodological differences. For example, Stürmer et al. (2013) measured pro-immigration attitudes, while Hodson et al. (2009) measured prejudice against immigrants. While these constructs are clearly related, they are also different, with one having a positive framing, the other more negative, which could account for the opposite direction of effect (see Table 1). Similarly, in terms of relationships between personality and social media use, Roos (2023) measured frequency of social media use, and found significant relationships with each of the Big Five personality traits, while others such as Lin et al. (2017) measured intensity of social media use and found different results.

Personality is considered relatively stable in adulthood (Bleidorn et al., 2022); however, opinions and social media use are subject to change. As such, if social media use interacts with the relationship between personality and opinions, changing social media use may provide another avenue to change opinions, either by encouraging the strengthening of more prosocial opinions, or by encouraging the weakening of antisocial opinions. This possible interaction needs to be investigated, which is the goal of the current study. The following hypotheses are proposed:

Hypothesis 1a: Based on Soutter et al. (2020) and Stürmer et al. (2013), it is predicted that each trait in the Big Five will positively predict favourable (progressive) opinions on at least one of the social issues of climate change, immigration, religious freedoms and gender equality, while each trait in the Dark Triad will negatively predict favourable opinions on those issues.

Hypothesis 1b: Based on Roos (2023) and Fox and Rooney (2015), it is predicted that time spent on social media will significantly contribute to the regression models tested in Hypothesis 1a.

**Exploratory Hypothesis 1c**: It is predicted that time spent on social media will moderate the relationships tested in Hypothesis 1a.

**Exploratory Hypothesis 2a**: It is predicted that personality will predict strength of opinion – regardless of direction – on at least one of climate change, immigration, religious freedoms, and gender equality.

**Exploratory Hypothesis 2b**: It is predicted that time spent on social media will significantly contribute to the regression model tested in Hypothesis 2a.

**Exploratory Hypothesis 2c**: It is predicted that time spent on social media will moderate the relationships tested in Hypothesis 2a.

## Method

### Participants

Psychology students (*N* = 536) participated in the study, with first-year students eligible to receive course credit. Ages ranged from 18 to 64 years old (*M* = 34.7, *SD* = 10.2). Further demographic characteristics are shown in Table 3.

**Table 3.** Demographic information for participants.

Demographic Variable	*n*	%
Gender		
Female	415	77.4
Male	116	21.6
Non-binary	3	0.6
Missing	2	0.4
Highest education level achieved		
Some high school	3	0.6
High school	41	7.6
Non-university post-school qualification	76	14.2
Some university	117	21.8
Undergraduate degree	190	35.4
Completed postgraduate degree	109	20.3
Annual household income		
Less than $20,000	28	5.2
More than $20,000 but less than $40,000	49	9.1
More than $40,000 but less than $60,000	58	10.8
More than $60,000 but less than $80,000	64	11.9
More than $80,000 but less than $100,000	74	13.8
More than $100,000 but less than $150,000	108	20.1
More than $150,000 but less than $200,000	61	11.4
More than $200,000	63	11.8
Prefer not to say	31	5.8
Political affiliation		
Liberal Party	133	24.8
Labor	137	25.6
Greens	120	22.4
Other/missing	146	27.2
Identifies as religious		
Yes	265	49.4
No	271	50.6

In Australia, of the political parties included in this table, the most progressive major party is the Greens, followed by Labor, with the Liberal Party the most conservative major party.

### Materials

Participants completed an anonymous online questionnaire hosted by Qualtrics (Provo, Utah). After providing demographic information (see Table 3), participants were asked to indicate their typical level of use of social media sites on a 9-point scale where 1 = *never*, 2 = *rarely*, 3 = *1–3 times per week*, 4 = *4–6 times per week*, 5 = *less than 1 hour per day*, 6 = *between 1 and 2 hours per day*, 7 = *between 2 and 3 hours per day*, 8 = *between 3 and 4 hours per day*, and 9 = *4+ hours per day.*

#### Personality scales

*Big Five Inventory (BFI; John & Srivastava, 1999)*. The BFI is a 44-question self-report inventory, scored from 1 (*disagree strongly*) to 5 (*agree strongly*). It contains subscales which measure the Big Five personality traits, with internal consistencies for each subscale ranging from α = .79 (agreeableness) to α = .88 (neuroticism; John & Srivastava, 1999). In the present study most α varied only marginally from the published values, with extraversion α = .86, agreeableness α = .79, neuroticism α = .86, and openness α = .77, except for conscientiousness, which varied to a greater degree, α = .65, compared to the published α = .82 (John & Srivastava, 1999).

*Short Dark Triad (SD3; Jones & Paulhus, 2014)*. The SD3 is a 27-question self-report inventory, scored from 1 (*disagree strongly*) to 5 (*agree strongly*), where each item is a statement designed to measure a participant’s level of Machiavellianism (α = .74–.76), narcissism (α = .68–.71), or psychopathy (α = .70, 9 items each; Jones & Paulhus, 2017). In the present study α varied only marginally from the published values, with Machiavellianism α = .80, narcissism α = .64, and psychopathy α = .69.

#### Opinion scales

Short scales were developed to assess opinions on each issue of interest using the ABC Vote Compass (see Appendix A; ABC, 2019). Each issue in the ABC Vote Compass was reviewed for suitability for inclusion in the present research. Of the 30 issues present in the ABC Vote Compass in (2019), nine were identified as suitable for inclusion. Four questions were developed to measure participant opinion on each issue. These questions were examined by each contributing researcher for suitability and face validity and adjusted as necessary. Each question was rated from 0 to 100, where 0 indicated *strongly disagree*, while 100 indicated *strongly agree*, and scale means were calculated. After data collection, all opinion scales were examined for performance, with five excluded due to either poor performance (e.g., low internal reliability or low completion rates) or lack of salience to current society (e.g., questions on COVID-19). The reliability of the remaining four opinion scales was found to range from adequate to excellent: Climate change α = .90; immigration α = .82; religious freedoms α = .62; and gender equality α = .65.

The four final opinion scale scores were combined to form a *progressive opinions* variable. To do this, the religious freedoms variable was reverse-coded (higher scores were initially indicative of more conservative opinions. This recoded variable was then used in all analyses), and then the mean of the four opinion scale scores was calculated. The internal consistency of this variable was found to be acceptable (α = .78).

Additionally, four *opinion strength* variables were calculated based on the strength of opinion, independent of direction, for each opinion scale. These variables were calculated by taking the distance from the neutral position (*x* = 50) for each opinion scale, for each participant. Opinion strength could therefore range from 0 to 50.

### Procedure

Students were recruited through online student message boards and university branded Facebook sites. These messages included a link directing participants to the Qualtrics questionnaire, which took approximately 20–30 minutes to complete. This study was approved by the UNE Human Research Ethics Committee (HE20–093).

### Data analysis

Participants who did not complete the personality scales, the time spent on social media question, and at least one opinion scale were excluded, yielding a total sample of *N* = 536. Other missing data within individual hierarchical multiple regressions were excluded listwise, resulting in varying sample sizes across each analysis. The variable “time spent on social media” was negatively skewed, with few responses for *never*, *rarely, 1–3 times per week*, and *4–6 times per week* (*n* < 20 for each). To overcome this, the variable was recoded from a 9-point Likert scale to a 6-point Likert scale. The 6-point scale combined *never, rarely, 1–3 times per week*, and *4–6 times per week* into one response, *less than once per day*. This resulted in a more normal distribution. Other assumptions for each hierarchical multiple regression were tested. No problematic univariate outliers were present, with all remaining scores across each variable conforming to the distribution pattern of the relevant histogram. Mahalanobis distance indicated the presence of multivariate outliers, however as Cook’s distance did not exceed 1 for any analysis (*D*_*max*_ = 0.19), indicating the multivariate outliers did not influence the analyses meaningfully, all data was retained. No other assumptions were violated.

## Results and discussion

Prior to completing hypothesis testing, the presence of potential covariates was investigated by analysing the correlations between all demographic, predictor and outcome variables. Table 4 shows those correlations, as well as descriptive statistics for each variable. All demographic variables were identified as potential covariates as each correlated with at least one personality variable. As such, each were added into the relevant hierarchical multiple-regression analyses (HMRA) at step 1 so that variance due to those demographic variables could be accounted for prior to drawing conclusions about the hypotheses.

**Table 4.** Descriptive statistics and intercorrelations for demographic variables, opinion variables, and personality variables.

Variable	1	2	3	4	5	6	7	8	9	10	11	12	13	14	15	16	17	18	19	20	21	22	23	24	25
1. Gender^b^	–																								
2. Age^p^	−.06	–																							
3. Political affiliation^s^	<.01	−.12*	–																						
4. Household Income^s^	.01	.29†	−.26†	–																					
5. Education Level^s^	−.03	.42†	.01	.20†	–																				
6. Identifies as religious^b^	.09*	.11*	−.25†	.03	.03	–																			
7. Extraversion^p^	.09*	.10*	−.12*	.10*	.05	.03	–																		
8. Neuroticism^p^	.07	−.22†	.16‡	−.15†	−.15†	−.04	−.35†	–																	
9. Agreeableness^p^	.19†	.05	−.02	−.04	−.02	.04	.18†	−.34†	–																
10. Conscientiousness^p^	.10*	.21†	−.19†	.16†	.15†	.09*	.24†	−.43†	.27†	–															
11. Openness^p^	−.05	.18†	.18†	−.04	.19†	−.05	.24†	−.11*	.08	.07	–														
12. Machiavellianism^p^	−.23†	−.05	−.05	<.01	<.01	−.08	−.11*	.17†	−.36†	−.14‡	−.10*	–													
13. Narcissism^p^	−.09*	<.01	−.04	.05	.04	.02	.47†	−.21†	−.04	.06	.18†	.20†	–												
14. Psychopathy^p^	−.28†	−.10*	.07	−.04	−.05	−.11‡	.04	.15†	−.46†	−.28†	−.01	.46†	.32†	–											
15. Time spent on social media^s^	.12‡	−.41†	.03	−.13‡	−.16†	−.01	−.07	.25†	−.07	−.23†	−.15†	.13‡	.03	.10*	–										
16. Climate Change: Direction^p^	.07	−.04	.48†	−.10*	.07	−.18†	.06	.11*	.02	−.07	.26†	−.03	−.05	−.01	−.03	–									
17. Climate Change: Strength^p^	−.02	−.02	.44†	−.10*	.07	−.16†	.05	.06	−.02	−.05	.24†	−.05	−.09*	.03	−.02	.65†	–								
18. Religious Freedoms: Direction^p^	.13‡	.04	.18†	.07	−.02	−.27†	.02	.07	.01	−.02	.07	−.05	−.09*	<.01	−.05	.42†	.24†	–							
19. Religious Freedoms: Strength^p^	−.04	.03	.12*	.04	−.02	−.10†	.01	.03	.08	.05	.07	.01	−.03	−.02	−.09*	.23†	.34†	.44†	–						
20. Immigration: Direction^p^	.13‡	−.10*	.45†	−.07	.04	−.17†	.04	.07	.17†	−.08	.18†	−.22†	−.03	−.16†	.11‡	.44†	.36†	.21†	.17†	–					
21. Immigration: Strength^p^	−.07	−.06	.37†	−.02	−.02	−.19†	.04	<.01	.06	−.02	.10*	−.05	.01	.01	.05	.27†	.44†	.18†	.41†	.49†	–				
22. Gender Equality: Direction^p^	.27†	.08	.28†	−.07	.02	−.12‡	.07	.10*	.11*	−.03	.16†	−.09*	−.04	−.08	.11*	.47†	.33†	.35†	.19†	.41†	.27†	–			
23. Gender Equality: Strength^p^	−.04	.05	.20†	−.04	−.06	−.10*	−.01	.08	.03	.01	.11*	−.03	−.09*	<.01	.10*	.23†	.45†	.16†	.34†	.19†	.40†	.45†	–		
24. Progressive Opinions: Direction^p^	.21†	−.01	.50†	−.08	.03	−.28†	.06	.12‡	.11*	−.06	.22†	−.14†	−.08	−.11*	.07	.79†	.53†	.68†	.35†	.72†	.42†	.76†	.35†	–	
25. Progressive Opinions: Strength^p^	.15†	<.01	.47†	−.08	.03	−.26†	<.01	.12‡	.05	−.07	.21†	−.15†	−.13‡	−.10*	.06	.58†	.66†	.52†	.44†	.65†	.57†	.62†	.53†	.80†	–
*n*	531	487	390	505	536	531	536	536	536	536	536	536	536	536	536	533	533	522	522	527	527	517	517	503	503
*M*	-	34.69	-	-	-	-	3.01	3.12	3.83	3.57	3.68	2.65	2.43	1.83	3.07	81.35	36.55	28.19	35.85	71.36	32.50	70.92	31.15	73.93	26.13
*SD*	-	10.21	-	-	-	-	0.82	0.82	0.60	0.51	0.58	0.65	0.53	0.53	1.47	21.17	12.68	22.95	12.18	23.82	12.56	22.42	13.29	16.61	12.86

* < .05.

‡< .01.

†< .001.

^b^relates to point-biserial correlations.

^s^relates to Spearman’s correlations.

^p^relates to Pearson’s bivariate correlations.

### Hypothesis 1

To test Hypotheses 1a, 1b, and 1c, relating to opinion direction, a series of HMRA were conducted. All demographic variables were entered at step 1, all personality traits were added at step 2, time spent on social media was added at step 3, and the interaction terms between time spent on social media and each personality trait were added at step 4. Step 1 of each of the HMRA show that the demographic factors as a whole significantly predicted some level of variance in opinion direction for each of the four issues, as well as progressive opinions, with *R*^2^ ranging from 11.3% (religious freedoms) to 28.2% (progressive opinions). Step 2 showed that the addition of personality variables significantly improved the predictive ability of the models for climate change, immigration, and progressive opinions, but not religious freedoms or gender equality. Tables 5 and 6 show the results for each of the HMRA.

**Table 5.** Model summary for opinion direction hierarchical regressions (H1).

Issue	Step	*F*	df	*p*	*R*^2^	∆*R*^2^	∆*p*
Climate Change	1	16.77	6, 325	<.001	.236		
	2	9.66	14, 317	<.001	.299	.063	<.001
	3	8.99	15, 316	<.001	.299	.000	.848
	4	5.96	23, 303	<.001	.308	.009	.866
Religious Freedoms	1	6.77	6, 320	<.001	.113		
	2	3.55	14, 312	<.001	.137	.025	.346
	3	3.33	15, 311	<.001	.138	.001	.575
	4	2.65	23, 308	<.001	.167	.029	.235
Immigration	1	13.15	6, 321	<.001	.197		
	2	8.52	14, 313	<.001	.276	.079	<.001
	3	8.51	15, 312	<.001	.290	.014	.012
	4	6.01	23, 304	<.001	.313	.022	.281
Gender Equality	1	9.38	6, 314	<.001	.152		
	2	4.85	14, 306	<.001	.182	.030	.200
	3	4.86	15, 305	<.001	.193	.011	.041
	4	3.53	23, 297	<.001	.215	.022	.408
Progressive Opinions	1	20.23	6, 309	<.001	.282		
	2	11.34	14, 301	<.001	.345	.063	<.001
	3	10.95	15, 300	<.001	.354	.009	.046
	4	7.31	23, 292	<.001	.365	.012	.723

**Table 6.** B and β coefficients, sr^2^ and p for opinion direction hierarchical regressions (H1).

Step	Variable	Climate Change			Religious Freedoms			Immigration			Gender Equality			Progressive Opinions		
		*B* [95% CI]	β	*sr*^2^	*B* [95% CI]	β	*sr*^2^	*B* [95% CI]	β	*sr*^2^	*B* [95% CI]	β	*sr*^2^	*B* [95% CI]	β	*sr*^2^
1	Gender	2.31 [−2.54, 7.15]	.05	<.01	4.33 [−1.25, 9.90]	.08	<.01	5.51 [−0.35, 11.36]	.09	.01	12.39 [6.75, 18.02]	.23	.05^†^	6.37 [2.68, 10.05]	.17	.03^†^
	Age	0.01 [−0.19, 0.21]	<.01	<.01	0.14 [−0.09, 0.38]	.07	<.01	0.07 [−0.17, 0.32]	.03	<.01	0.37 [0.14, 0.61]	.18	.03^‡^	0.15 [−0.01, 0.30]	.10	.01
	Political affiliation	11.39 [8.92, 13.87]	.47	.19^†^	4.56 [1.70, 7.42]	.18	.03^‡^	11.18 [8.18, 14.18]	.39	.13^†^	7.34 [4.44, 10.24]	.28	.07^†^	8.47 [6.57, 10.38]	.45	.18^†^
	Household Income	0.69 [−0.35, 1.72]	.07	<.01	1.17 [−0.02, 2.36]	.11	.01	0.07 [−1.18, 1.32]	.01	<.01	−0.40 [−1.61, 0.81]	−.04	<.01	0.31 [−0.49, 1.11]	.04	<.01
	Education	0.78 [−0.95, 2.50]	.05	<.01	−2.18 [−4.17, −0.18]	−.12	.01*	2.17 [0.08, 4.26]	.11	.01*	0.04 [−1.99, 2.06]	<.01	<.01	0.27 [−1.06, 1.60]	.02	<.01
	Identifies as religious	−3.40 [−7.28, 0.48]	−.09	.01	−9.37 [−13.87, −4.87]	−.23	.05^†^	−5.34 [−10.04, −0.64]	−.12	.01*	−3.90 [−8.46, 0.65]	−.09	.01	−5.65 [−8.65, −2.64]	−.19	.03^†^
2	Gender	1.95 [−3.09, 6.99]	.04	<.01	2.92 [−3.08, 8.92]	.05	<.01	2.18 [−3.86, 8.23]	.04	<.01	11.71 [5.68, 17.74]	.22	.04^†^	4.88 [1.05, 8.72]	.13	.01*
	Age	−0.05 [−0.25, 0.16]	−.03	<.01	0.12 [−0.12, 0.36]	.06	<.01	0.01 [−0.23, 0.25]	<.01	<.01	0.30 [0.05, 0.54]	.14	.02*	0.09 [−0.07, 0.25]	.06	<.01
	Political affiliation	10.26 [7.74, 12.78]	.42	.14^†^	4.12 [1.13, 7.11]	.16	.02^‡^	9.66 [6.64, 12.68]	.34	.09^†^	6.74 [3.73, 9.75]	.25	.05^†^	7.63 [5.71, 9.55]	.41	.13^†^
	Household Income	0.71 [−0.32, 1.73]	.07	<.01	1.27 [0.05, 2.49]	.12	.01*	0.42 [−0.81, 1.66]	.04	<.01	−0.26 [−1.50, 0.97]	−.02	<.01	0.48 [−0.32, 1.27]	.06	<.01
	Education	0.42 [−1.29, 2.14]	.03	<.01	−2.06 [−4.10, −0.03]	−.11	.01*	2.25 [0.20, 4.31]	.11	.01*	−0.27 [−2.33, 1.78]	−.01	<.01	0.14 [−1.17, 1.45]	.01	<.01
	Identifies as religious	−3.49 [−7.27, 0.29]	−.09	.01	−9.66 [−14.17, −5.15]	−.23	.05^†^	−5.66 [−10.20, −1.13]	−.12	.01*	−3.75 [−8.31, 0.80]	−.09	.01	−5.70 [−8.62, −2.78]	−.19	.03^†^
	Extraversion	3.83 [1.12, 6.55]	.17	.02^‡^	1.66 [−1.60, 4.92]	.07	<.01	1.64 [−1.64, 4.92]	.06	<.01	2.08 [−1.20, 5.36]	.08	<.01	2.18 [0.06, 4.30]	.12	.01*
	Neuroticism	0.79 [−1.85, 3.43]	.03	<.01	1.42 [−1.70, 4.55]	.06	<.01	2.09 [−1.08, 5.25]	.08	<.01	0.48 [−2.68, 3.64]	.02	<.01	1.18 [−0.84, 3.19]	.07	<.01
	Agreeableness	−1.51 [−5.26, 2.23]	−.05	<.01	3.26 [−1.21, 7.73]	.09	.01	0.44 [−4.08, 4.97]	.01	<.01	1.78 [−2.67, 6.24]	.05	<.01	1.13 [−1.75, 4.01]	.05	<.01
	Conscientiousness	−2.01 [−6.28, 2.25]	−.05	<.01	1.01 [−4.16, 6.18]	.02	<.01	−4.88 [−10.03, 0.26]	−.10	.01	−1.05 [−6.24, 4.13]	−.02	<.01	−1.53 [−4.87, 1.81]	−.05	<.01
	Openness	6.19 [2.67, 9.70]	.18	.03^†^	1.44 [−2.75, 5.63]	.04	<.01	3.84 [−0.36, 8.04]	.10	.01	4.98 [0.76, 9.20]	.13	.01*	3.98 [1.29, 6.66]	.15	.02^‡^
	Machiavellianism	0.85 [−2.46, 4.16]	.03	<.01	−2.59 [−6.53, 1.35]	−.08	.01	−7.42 [−11.39, −3.44]	−.21	.03^†^	0.40 [−3.54, 4.33]	.01	<.01	−2.41 [−4.94, 0.13]	−.10	.01
	Narcissism	−7.47 [−11.91, −3.03]	−.20	.02^†^	−3.03 [−8.33, 2.28]	−.08	<.01	1.48 [−3.86, 6.81]	.03	<.01	−1.98 [−7.27, 3.31]	−.05	<.01	−2.55 [−5.95, 0.85]	−.09	.01
	Psychopathy	−1.89 [−6.38, 2.59]	−.05	<.01	3.26 [−2.21, 8.74]	.08	<.01	−3.44 [−8.91, 2.03]	−.08	<.01	−1.94 [−7.37, 3.50]	−.05	<.01	−0.87 [−4.38, 2.64]	−.03	<.01
3	Gender	2.02 [−3.08, 7.13]	.04	<.01	2.66 [−3.42, 8.74]	.05	<.01	1.01 [−5.05, 7.08]	.02	<.01	10.77 [4.70, 16.83]	.20	.03^†^	4.26 [0.40, 8.12]	.11	.01*
	Age	−0.05 [−0.27, 0.16]	−.03	<.01	0.14 [−0.11, 0.40]	.07	<.01	0.12 [−0.14, 0.37]	.05	<.01	0.39 [0.13, 0.64]	.18	.02^‡^	0.15 [−0.02, 0.31]	.10	.01
	Political affiliation	10.25 [7.72, 12.78]	.42	.14^†^	4.17 [1.17, 7.17]	.16	.02^‡^	9.89 [6.89, 12.89]	.34	.10^†^	6.92 [3.92, 9.92]	.26	.06^†^	7.72 [5.81, 9.63]	.41	.14^†^
	Household Income	0.70 [−0.33, 1.73]	.07	<.01	1.29 [0.07, 2.51]	.12	.01*	0.48 [−0.74, 1.71]	.04	<.01	−0.19 [−1.42, 1.05]	−.02	<.01	0.52 [−0.27, 1.31]	.07	<.01
	Education	0.43 [−1.29, 2.15]	.03	<.01	−2.10 [−4.14, −0.06]	−.12	.01*	2.07 [0.02, 4.11]	.10	.01*	−0.44 [−2.49, 1.61]	−.02	<.01	0.04 [−1.27, 1.35]	<.01	<.01
	Identifies as religious	−3.49 [−7.27, 0.30]	−.09	.01	−9.69 [−14.20, −5.17]	−.23	.05^†^	−5.68 [−10.18, −1.18]	−.12	.01*	−3.83 [−8.36, 0.70]	−.09	.01	−5.80 [−8.70, −2.89]	−.19	.03^†^
	Extraversion	3.84 [1.12, 6.55]	.17	.02^‡^	1.65 [−1.62, 4.91]	.07	<.01	1.64 [−1.62, 4.89]	.06	<.01	2.04 [−1.23, 5.30]	.08	<.01	2.15 [0.04, 4.27]	.12	.01*
	Neuroticism	0.84 [−1.85, 3.53]	.04	<.01	1.26 [−1.92, 4.44]	.05	<.01	1.34 [−1.85, 4.53]	.05	<.01	−0.04 [−3.22, 3.15]	<.01	<.01	0.84 [−1.19, 2.87]	.05	<.01
	Agreeableness	−1.49 [−5.25, 2.28]	−.05	<.01	3.14 [−1.35, 7.64]	.09	.01	−0.07 [−4.57, 4.44]	<.01	<.01	1.40 [−3.05, 5.85]	.04	<.01	0.82 [−2.06, 3.71]	.03	<.01
	Conscientiousness	−2.04 [−6.33, 2.24]	−.05	<.01	1.12 [−4.07, 6.30]	.03	<.01	−4.48 [−9.59, 0.64]	−.10	.01	−0.68 [−5.85, 4.49]	−.02	<.01	−1.25 [−4.59, 2.08]	−.04	<.01
	Openness	6.17 [2.65, 9.69]	.18	.03^†^	1.50 [−2.70, 5.70]	.04	<.01	4.09 [−0.08, 8.27]	.10	.01	5.15 [0.95, 9.35]	.14	.02*	4.09 [1.42, 6.76]	.16	.02^‡^
	Machiavellianism	0.86 [−2.45, 4.18]	.03	<.01	−2.64 [−6.58, 1.30]	−.08	.01	−7.56 [−11.50, −3.62]	−.22	.03^†^	0.28 [−3.64, 4.20]	.01	<.01	−2.48 [−5.00, 0.05]	−.11	.01
	Narcissism	−7.45 [−11.90, −2.99]	−.20	.02^†^	−3.10 [−8.41, 2.22]	−.08	<.01	1.08 [−4.22, 6.38]	.02	<.01	−2.31 [−7.59, 2.96]	−.06	<.01	−2.74 [−6.13, 0.65]	−.10	.01
	Psychopathy	−1.87 [−6.37, 2.63]	−.05	<.01	3.16 [−2.33, 8.65]	.08	<.01	−3.84 [−9.28, 1.59]	−.09	<.01	−2.28 [−7.70, 3.13]	−.06	<.01	−1.14 [−4.64, 2.37]	−.04	<.01
	SM	−0.13 [−1.51, 1.24]	−.01	<.01	0.47 [−1.18, 2.12]	.03	<.01	2.12 [0.47, 3.77]	.14	.01*	1.73 [0.07, 3.39]	.12	.01*	1.09 [0.02, 2.16]	.11	.01*
4	Gender	1.70 [−3.52, 6.92]	.03	<.01	1.33 [−4.82, 7.48]	.02	<.01	1.03 [−5.11, 7.17]	.02	<.01	11.42 [5.26, 17.58]	.21	.04^†^	4.02 [0.08, 7.96]	.10	.01*
	Age	−0.05 [−0.27, 0.17]	−.03	<.01	0.16 [−0.10, 0.42]	.08	<.01	0.09 [−0.17, 0.35]	.04	<.01	0.38 [0.12, 0.64]	.18	.02^‡^	0.14 [−0.03, 0.31]	.10	.01
	Political affiliation	10.31 [7.73, 12.88]	.42	.14^†^	4.29 [1.27, 7.31]	.17	.02^‡^	9.83 [6.80, 12.85]	.34	.09^†^	6.78 [3.75, 9.81]	.25	.05^†^	7.70 [5.76, 9.64]	.41	.13^†^
	Household Income	0.65 [−0.41, 1.70]	.06	<.01	1.13 [−0.11, 2.37]	.11	.01	0.50 [−0.74, 1.75]	.04	<.01	−0.28 [−1.54, 0.97]	−.03	<.01	0.44 [−0.37, 1.25]	.06	<.01
	Education	0.20 [−1.57, 1.98]	.01	<.01	−2.01 [−4.09, 0.07]	−.11	.01	2.49 [0.41, 4.58]	.13	.01*	−0.46 [−2.56, 1.64]	−.02	<.01	0.14 [−1.21, 1.48]	.01	<.01
	Identifies as religious	−3.39 [−7.28, 0.51]	−.09	.01	−8.86 [−13.46, −4.26]	−.21	.04^†^	−5.22 [−9.81, −0.62]	−.11	.01*	−4.51 [−9.13, 0.12]	−.11	.01	−5.65 [−8.64, −2.66]	−.19	.03^†^
	Extraversion	4.39 [1.55, 7.24]	.19	.02^‡^	2.54 [−0.85, 5.92]	.11	.01	1.50 [−1.87, 4.86]	.06	<.01	1.99 [−1.41, 5.38]	.08	<.01	2.50 [0.30, 4.71]	.14	.01*
	Neuroticism	0.61 [−2.14, 3.37]	.03	<.01	1.17 [−2.06, 4.39]	.05	<.01	1.27 [−1.97, 4.50]	.05	<.01	0.20 [−3.05, 3.45]	.01	<.01	0.76 [−1.32, 2.84]	.04	<.01
	Agreeableness	−1.79 [−5.69, 2.11]	−.06	<.01	3.38 [−1.23, 7.99]	.10	.01*	0.82 [−3.79, 5.43]	.02	<.01	1.20 [−3.38, 5.78]	.03	<.01	0.95 [−2.02, 3.91]	.04	<.01
	Conscientiousness	−2.20 [−6.55, 2.14]	−.06	<.01	0.20 [−5.02, 5.42]	<.01	<.01	−4.51 [−9.66, 0.65]	−.10	.01	−0.66 [−5.91, 4.58]	−.02	<.01	−1.55 [−4.95, 1.84]	−.05	<.01
	Openness	6.08 [2.51, 9.65]	.18	.03^†^	0.89 [−3.32, 5.11]	.02	<.01	3.96 [−0.23, 8.15]	.10	.01	5.71 [1.48, 9.95]	.15	.02^‡^	4.00 [1.29, 6.71]	.15	.02^‡^
	Machiavellianism	0.71 [−2.72, 4.14]	.02	<.01	−3.12 [−7.15, 0.92]	−.10	.01	−6.66 [−10.70, −2.63]	−.19	.02^†^	0.40 [−3.64, 4.45]	.01	<.01	−2.37 [−4.98, 0.24]	−.10	.01
	Narcissism	−7.88 [−12.62, −3.14]	−.21	.02^†^	−2.87 [−8.45, 2.71]	−.07	<.01	0.82 [−4.76, 6.39]	.02	<.01	−3.02 [−8.59, 2.56]	−.07	<.01	−3.05 [−6.63, 0.53]	−.11	.01
	Psychopathy	−1.81 [−6.41, 2.78]	−.05	<.01	2.77 [−2.77, 8.32]	.07	<.01	−3.87 [−9.35, 1.62]	−.09	<.01	−2.74 [−8.22, 2.75]	−.07	<.01	−1.40 [−4.97, 2.16]	−.05	<.01
	SM	−0.05 [−1.47, 1.37]	<.01	<.01	0.56 [−1.13, 2.24]	.04	<.01	2.02 [0.33, 3.71]	.13	.01*	1.27 [−0.43, 2.96]	.09	.01	0.99 [−0.11, 2.09]	.10	.01
	SMxExtraversion	0.49 [−1.28, 2.26]	.04	<.01	−0.66 [−2.73, 1.41]	−.05	<.01	0.22 [−1.87, 2.31]	.01	<.01	1.83 [−0.26, 3.92]	.12	.01	0.55 [−0.80, 1.91]	.05	<.01
	SMxNeuroticism	−0.24 [−2.14, 1.66]	−.02	<.01	−0.90 [−3.15, 1.34]	−.06	<.01	0.34 [−1.90, 2.59]	.02	<.01	1.53 [−0.78, 3.83]	.09	<.01	0.18 [−1.29, 1.65]	.02	<.01
	SMxAgreeableness	−1.62 [−4.25, 1.01]	−.08	<.01	−3.30 [−6.41, −0.20]	−.16	.01	1.33 [−1.78, 4.43]	.06	<.01	0.42 [−2.66, 3.50]	.02	<.01	−0.87 [−2.87, 1.12]	−.06	<.01
	SMxConscientiousness	−0.63 [−3.62, 2.35]	−.03	<.01	−1.54 [−5.22, 2.13]	−.05	<.01	−0.16 [−3.83, 3.50]	−.01	<.01	1.46 [−2.40, 5.32]	.05	<.01	−0.44 [−2.91, 2.04]	−.02	<.01
	SMxOpenness	0.78 [−1.51, 3.08]	.03	<.01	1.87 [−0.84, 4.58]	.08	.01	−0.54 [−3.25, 2.16]	−.02	<.01	−1.16 [−3.90, 1.59]	−.05	<.01	0.22 [−1.55, 1.98]	.01	<.01
	SMxMachiavellianism	0.41 [−1.90, 2.72]	.02	<.01	−0.71 [−3.42, 1.99]	−.03	<.01	2.62 [−0.10, 5.35]	.11	.01	−1.59 [−4.32, 1.15]	−.07	<.01	0.30 [−1.46, 2.06]	.02	<.01
	SMxNarcissism	−1.29 [−4.32, 1.73]	−.05	<.01	−1.30 [−4.84, 2.25]	−.05	<.01	−0.42 [−3.98, 3.14]	−.01	<.01	−1.29 [−4.84, 2.26]	−.05	<.01	−1.21 [−3.49, 1.06]	−.07	<.01
	SMxPsychopathy	−2.06 [−5.08, 0.96]	−.09	<.01	0.26 [−3.32, 3.84]	.01	<.01	2.67 [−0.90, 6.24]	.10	.01	2.76 [−0.82, 6.34]	.11	.01	0.85 [−1.46, 3.15]	.05	<.01

SM = Time spent on social media.

*< .05.

‡< .01.

†< .001.

When considering individual predictors, political affiliation was found to be a significant predictor at each step of each HMRA. As each of the topics were chosen based on the ABC Vote Compass, a political reference tool, these results are not surprising. Many other significant predictor variables were also as expected, such as gender significantly predicting opinions on gender equality, and identifying as religious significantly predicting opinions on religious freedoms.

Hypothesis 1a was partially supported by step 2 of the analyses, with several personality traits significantly predicting opinion direction for climate change (extraversion *sr*^2^ = .017, openness *sr*^2^ = .027, and narcissism *sr*^2^ = .024), immigration (Machiavellianism *sr*^2^ = .031), gender equality (openness *sr*^2^ = .014), and progressive opinions (extraversion *sr*^2^ = .009, openness *sr*^2^ = .019).

Regarding the findings concerning opinion direction for climate change and extraversion, openness and narcissism align with past research, which found that people higher in extraversion and openness tend to have more pro-environmental views, and those higher in narcissism tend to have less pro-environmental views (e.g., Annalakshmi et al., 2020; Soutter et al., 2020; Stürmer et al., 2013). Although the non-significant findings relating to the ability of agreeableness, conscientiousness, neuroticism, Machiavellianism and psychopathy to predict climate change opinions did not match previous research, these variables are not always found to be significant predictors of pro-environmental views. This suggests that some differences in either the methodology or the sample characteristics may account for these different results. For example, the short surveys used to measure each of the issues presented in this research were designed for this study, making them different to past research.

Different conceptualisations of personality traits, such as using the HEXACO rather than a scale based on the Big Five (e.g., Annalakshmi et al., 2020; Stürmer et al., 2013) may also have played a role. Although the broad traits are similar across the HEXACO and the BFI, there are some differences, notably relating to agreeableness (Ashton & Lee, 2008). These explanations may also apply to the different results relating to opinions on immigration in this study when compared to past research (e.g., Arvan, 2013; Stürmer et al., 2013).

An alternate explanation for the different findings is the different populations used in each study; that is, relationships between personality and opinion may be different for Australians compared to other countries. Given past research shows that opinions on both climate change and immigration vary by country (e.g., Skamp et al., 2021; Ueffing et al., 2015), this may explain the results found in the current study. It may also explain why the only significant predictor in the model for opinions on immigration was Machiavellianism, despite expected relationships between those opinions and openness, extraversion, agreeableness, narcissism and psychopathy.

The overall models for religious freedoms and gender equality were non-significant at step 2, although openness was still found to significantly predict variance in opinions on gender equality (*sr*^2^ = .014). In this study, higher scores on opinions about gender equality suggested more progressive views. Given this study found a positive correlation between openness and political affiliation (where higher political affiliation relates to supporting more progressive political parties), and previous studies that have found negative correlations between openness and general conservatism (van Hiel & Mervielde, 2004; van Hiel et al., 2000), these results are not surprising.

Finally, when exploring the overall progressive opinions variable, gender, political affiliation, and identifying as religious were all significant at both steps 1 and 2 of the HMRA. Extraversion and openness were both significant at step 2 of the HMRA, suggesting that those higher in each of those traits were more likely to express more progressive opinions overall. Given the research into both climate change and immigration identifies openness and extraversion as significant predictors of progressive opinions on those issues, this is not surprising, and suggests that they are both generally related to progressive opinions.

Hypothesis 1b was partially supported. The addition of time spent on social media at step 3 significantly improved the predictive ability of the models for immigration (∆*R*^2^ = .014), gender equality (∆*R*^2^ = .011), and progressive opinions (∆*R*^2^ = .009), but not climate change or religious freedoms. Hypothesis 1c, exploring whether time spent on social media would moderate the relationship between personality and opinion direction on each issue, was not supported. There was no significant improvement at step 4 of any of the models, suggesting there is no moderating effect of time spent on social media on the relationship between opinion direction and personality. This suggests that although personality traits and time spent on social media combine to predict opinion direction on several social issues, time spent on social media does not affect the strength of the relationship between personality traits and those opinions, and each relationship is independent of each other. As such, although the amount of time an individual spends on social media alone may contribute to more progressive or conservative views on a topic, individuals higher in particular personality traits are not at risk of polarising further than other individuals.

### Hypothesis 2

To test Hypotheses 2a, 2b, and 2c, relating to opinion strength – regardless of direction – a series of HMRA were conducted. Tables 7 and 8 show the results of these analyses. Each step was structured in the same manner as the first set of HMRA. Step 1 of each of the HMRA show that the demographic factors as a whole significantly predicted variance in opinion strength for climate change, immigration, gender equality, and progressive opinions, with *R*^2^ ranging from 8.1% (gender equality) to 25.8% (progressive opinions).

**Table 7.** Model summary for opinion strength hierarchical regressions (H2).

Issue	Step	*F*	df	*p*	*R*^2^	∆*R*^2^	∆*p*
Climate Change	1	15.66	6, 325	<.001	.224		
	2	9.70	14, 317	<.001	.300	.076	<.001
	3	9.03	15, 316	<.001	.300	.000	.816
	4	6.07	23, 308	<.001	.312	.012	.720
Religious Freedoms	1	1.21	6, 320	.301	.022		
	2	0.99	14, 312	.461	.043	.020	.573
	3	1.07	15, 311	.386	.049	.006	.150
	4	1.19	23, 303	.248	.083	.034	.192
Immigration	1	10.25	6, 321	<.001	.161		
	2	4.72	14, 313	<.001	.174	.014	.740
	3	4.47	15, 312	<.001	.177	.003	.321
	4	3.49	23, 304	<.001	.209	.032	.143
Gender Equality	1	4.61	6, 314	<.001	.081		
	2	2.71	14, 306	<.001	.110	.030	.259
	3	2.91	15, 305	<.001	.125	.015	.023
	4	2.44	23, 297	<.001	.159	.033	.166
Progressive Opinions	1	17.88	6, 309	<.001	.258		
	2	9.57	14, 301	<.001	.308	.050	.006
	3	9.18	15, 300	<.001	.315	.007	.087
	4	6.62	23, 292	<.001	.343	.028	.140

**Table 8.** B and β coefficients, sr^2^ and p for opinion strength hierarchical regressions (H2).

Step	Variable	Climate Change			Religious Freedoms			Immigration			Gender Equality			Progressive Opinions		
		*B* [95% CI]	β	*sr*^2^	*B* [95% CI]	β	*sr*^2^	*B* [95% CI]	β	*sr*^2^	*B* [95% CI]	β	*sr*^2^	*B* [95% CI]	β	*sr*^2^
1	Gender	1.39 [−1.72, 4.51]	.04	<.01	0.00 [−3.19, 3.19]	<.01	<.01	−1.22 [−4.39, 1.95]	−.04	<.01	0.55 [−3.15, 4.25]	.02	<.01	5.08 [1.86, 8.30]	.15	.02^‡^
	Age	0.04 [−0.09, 0.17]	.03	<.01	0.02 [−0.11, 0.16]	.02	<.01	0.01 [−0.12, 0.14]	.01	<.01	0.19 [0.04, 0.35]	.15	.02*	0.15 [0.02, 0.29]	.12	.01*
	Political affiliation	6.97 [5.38, 8.56]	.45	.18^†^	1.58 [−0.06, 3.22]	.11	.01	5.45 [3.82, 7.08]	.36	.11^†^	4.10 [2.19, 6.00]	.24	.05^†^	7.16 [5.50, 8.83]	.44	.17^†^
	Household Income	0.14 [−0.53, 0.80]	.02	<.01	0.21 [−0.47, 0.89]	.04	<.01	0.35 [−0.32, 1.03]	.06	<.01	0.23 [−0.56, 1.03]	.03	<.01	0.35 [−0.35, 1.04]	.05	<.01
	Education	0.68 [−0.43, 1.79]	.06	<.01	−0.39 [−1.53, 0.75]	−.04	<.01	0.64 [−0.49, 1.78]	.06	<.01	−0.87 [−2.20, 0.46]	−.07	.01	0.25 [−0.91, 1.41]	.02	<.01
	Identifies as religious	−2.15 [−4.64, 0.34]	−.09	.01	−1.75 [−4.32, 0.83]	−.08	.01	−2.69 [−5.24, −0.14]	−.11	.01*	−2.14 [−5.13, 0.86]	−.08	.01	−4.06 [−6.68, −1.43]	−.16	.02^‡^
2	Gender	2.11 [−1.10, 5.32]	.07	<.01	−0.48 [−3.92, 2.97]	−.02	<.01	−1.74 [−5.16, 1.69]	−.06	<.01	0.08 [−3.89, 4.05]	<.01	<.01	4.24 [0.85, 7.63]	.13	.01*
	Age	0.02 [−0.11, 0.15]	.02	<.01	0.01 [−0.13, 0.15]	.01	<.01	−0.01 [−0.15, 0.13]	−.01	<.01	0.16 [0.00, 0.32]	.12	.01	0.12 [−0.01, 0.26]	.10	.01
	Political affiliation	6.04 [4.43, 7.64]	.39	.12^†^	1.54 [−0.18, 3.25]	.11	.01	5.25 [3.54, 6.95]	.34	.10^†^	3.70 [1.72, 5.68]	.22	.04^†^	6.30 [4.61, 8.00]	.39	.12^†^
	Household Income	0.12 [−0.53, 0.78]	.02	<.01	0.25 [−0.45, 0.95]	.04	<.01	0.31 [−0.39, 1.01]	.05	<.01	0.30 [−0.52, 1.11]	.04	<.01	0.50 [−0.20, 1.20]	.07	<.01
	Education	0.48 [−0.61, 1.57]	.04	<.01	−0.36 [−1.53, 0.81]	−.04	<.01	0.55 [−0.61, 1.72]	.05	<.01	−0.96 [−2.31, 0.40]	−.08	.01	0.16 [−0.99, 1.32]	.01	<.01
	Identifies as religious	−2.20 [−4.60, 0.21]	−.09	.01	−1.79 [−4.38, 0.80]	−.08	.01	−2.77 [−5.34, −0.20]	−.11	.01*	−2.33 [−5.33, 0.67]	−.09	.01	−4.10 [−6.68, −1.52]	−.16	.02^‡^
	Extraversion	2.20 [0.47, 3.93]	.15	.01*	0.98 [−0.89, 2.85]	.07	<.01	1.22 [−0.63, 3.08]	.09	<.01	0.64 [−1.52, 2.80]	.04	<.01	1.09 [−0.79, 2.96]	.07	<.01
	Neuroticism	0.18 [−1.50, 1.86]	.01	<.01	0.84 [−0.96, 2.63]	.06	<.01	0.14 [−1.65, 1.93]	.01	<.01	1.34 [−0.74, 3.42]	.08	.01	0.98 [−0.80, 2.75]	.06	<.01
	Agreeableness	−1.18 [−3.57, 1.21]	−.06	<.01	2.21 [−0.36, 4.78]	.12	.01	−1.23 [−3.79, 1.33]	−.06	<.01	1.36 [−1.58, 4.29]	.06	<.01	−0.37 [−2.92, 2.17]	−.02	<.01
	Conscientiousness	−1.22 [−3.94, 1.49]	−.05	<.01	0.73 [−2.23, 3.70]	.03	<.01	0.19 [−2.73, 3.10]	.01	<.01	2.48 [−0.93, 5.89]	.09	.01	−1.95 [−4.90, 1.00]	−.07	<.01
	Openness	4.38 [2.14, 6.61]	.20	.03^†^	0.78 [−1.63, 3.18]	.04	<.01	1.20 [−1.18, 3.58]	.06	<.01	2.65 [−0.13, 5.42]	.11	.01	3.13 [0.76, 5.51]	.14	.02^‡^
	Machiavellianism	−0.18 [−2.28, 1.93]	−.01	<.01	0.64 [−1.62, 2.90]	.04	<.01	−0.84 [−3.09, 1.41]	−.05	<.01	−0.88 [−3.47, 1.71]	−.04	<.01	−2.08 [−4.33, 0.16]	−.10	.01
	Narcissism	−5.87 [−8.70, −3.04]	−.25	.04^†^	−1.95 [−4.99, 1.10]	−.09	.01	−0.90 [−3.92, 2.12]	−.04	<.01	−1.63 [−5.11, 1.85]	−.06	<.01	−2.49 [−5.50, 0.51]	−.10	.01
	Psychopathy	2.18 [−0.67, 5.04]	.09	.01	0.83 [−2.31, 3.97]	.04	<.01	−0.89 [−3.98, 2.21]	−.04	<.01	1.49 [−2.09, 5.06]	.06	<.01	−0.83 [−3.93, 2.28]	−.03	<.01
3	Gender	2.05 [−1.20, 5.30]	.06	<.01	−0.09 [−3.57, 3.39]	<.01	<.01	−2.00 [−5.46, 1.46]	−.06	<.01	−0.61 [−4.60, 3.38]	−.02	<.01	3.77 [0.35, 7.19]	.11	.01*
	Age	0.02 [−0.11, 0.16]	.02	<.01	−0.02 [−0.17, 0.12]	−.02	<.01	0.02 [−0.13, 0.16]	.01	<.01	0.23 [0.06, 0.39]	.17	.02^‡^	0.17 [0.02, 0.31]	.13	.01*
	Political affiliation	6.05 [4.44, 7.66]	.39	.12^†^	1.47 [−0.25, 3.18]	.10	.01	5.30 [3.58, 7.01]	.35	.10^†^	3.83 [1.86, 5.80]	.23	.04^†^	6.37 [4.68, 8.07]	.39	.13^†^
	Household Income	0.13 [−0.53, 0.78]	.02	<.01	0.23 [−0.47, 0.93]	.04	<.01	0.33 [−0.37, 1.03]	.05	<.01	0.35 [−0.46, 1.16]	.05	<.01	0.53 [−0.17, 1.23]	.08	.01
	Education	0.47 [−0.63, 1.56]	.04	<.01	−0.31 [−1.47, 0.86]	−.03	<.01	0.51 [−0.66, 1.68]	.05	<.01	−1.08 [−2.42, 0.27]	−.09	.01	0.09 [−1.07, 1.25]	.01	<.01
	Identifies as religious	−2.20 [−4.61, 0.21]	−.09	.01	−1.76 [−4.35, 0.83]	−.08	.01	−2.77 [−5.34, −0.20]	−.11	.01*	−2.39 [−5.37, 0.59]	−.09	.01	−4.17 [−6.75, −1.60]	−.16	.02^‡^
	Extraversion	2.20 [0.47, 3.93]	.15	.01*	1.01 [−0.86, 2.87]	.08	<.01	1.22 [−0.63, 3.08]	.09	<.01	0.61 [−1.54, 2.75]	.04	<.01	1.07 [−0.80, 2.94]	.07	<.01
	Neuroticism	0.14 [−1.57, 1.85]	.01	<.01	1.08 [−0.74, 2.90]	.08	<.01	−0.03 [−1.85, 1.80]	<.01	<.01	0.96 [−1.13, 3.05]	.06	<.01	0.72 [−1.07, 2.52]	.05	<.01
	Agreeableness	−1.20 [−3.60, 1.20]	−.06	<.01	2.38 [−0.19, 4.96]	.13	.01	−1.34 [−3.91, 1.23]	−.07	<.01	1.07 [−1.85, 4.00]	.05	<.01	−0.60 [−3.16, 1.95]	−.03	<.01
	Conscientiousness	−1.20 [−3.92, 1.53]	−.05	<.01	0.57 [−2.40, 3.54]	.02	<.01	0.28 [−2.64, 3.19]	.01	<.01	2.76 [−0.64, 6.15]	.10	.01	−1.74 [−4.69, 1.21]	−.06	<.01
	Openness	4.39 [2.15, 6.63]	.20	.03^†^	0.69 [−1.72, 3.09]	.03	<.01	1.26 [−1.12, 3.64]	.06	<.01	2.77 [0.01, 5.53]	.12	.01*	3.22 [0.85, 5.59]	.14	.02^‡^
	Machiavellianism	−0.19 [−2.30, 1.92]	−.01	<.01	0.71 [−1.54, 2.97]	.04	<.01	−0.87 [−3.12, 1.38]	−.05	<.01	−0.97 [−3.54, 1.61]	−.05	<.01	−2.14 [−4.37, 0.09]	−.11	.01
	Narcissism	−5.89 [−8.73, −3.06]	−.25	.04^†^	−1.85 [−4.89, 1.19]	−.09	<.01	−0.99 [−4.01, 2.04]	−.04	<.01	−1.88 [−5.34, 1.59]	−.07	<.01	−2.64 [−5.64, 0.36]	−.11	.01
	Psychopathy	2.17 [−0.70, 5.03]	.09	.01	0.98 [−2.17, 4.12]	.04	<.01	−0.98 [−4.08, 2.12]	−.04	<.01	1.23 [−2.33, 4.79]	.05	<.01	−1.03 [−4.13, 2.08]	−.04	<.01
	Time on Social Media	0.10 [−0.77, 0.98]	.01	<.01	−0.69 [−1.64, 0.25]	−.09	.01	0.48 [−0.47, 1.42]	.06	<.01	1.26 [0.17, 2.35]	.14	.02*	0.83 [−0.12, 1.77]	.09	.01
4	Gender	2.12 [−1.20, 5.43]	.07	<.01	−0.47 [−3.99, 3.05]	−.02	<.01	−1.88 [−5.37, 1.61]	−.06	<.01	−0.45 [−4.48, 3.57]	−.01	<.01	3.68 [0.23, 7.13]	.11	.01*
	Age	0.00 [−0.14, 0.14]	<.01	<.01	−0.03 [−0.18, 0.12]	−.03	<.01	0.01 [−0.14, 0.16]	.01	<.01	0.23 [0.06, 0.41]	.18	.02^‡^	0.16 [0.01, 0.31]	.12	.01*
	Political affiliation	6.01 [4.38, 7.65]	.39	.12^†^	1.74 [0.01, 3.46]	.12	.012*	5.48 [3.76, 7.20]	.36	.10^†^	4.00 [2.02, 5.98]	.24	.05^†^	6.28 [4.58, 7.98]	.39	.12^†^
	Household Income	0.14 [−0.53, 0.82]	.02	<.01	0.23 [−0.48, 0.93]	.04	<.01	0.29 [−0.41, 1.00]	.05	<.01	0.22 [−0.60, 1.04]	.03	<.01	0.47 [−0.24, 1.18]	.07	<.01
	Education	0.49 [−0.64, 1.61]	.05	<.01	−0.14 [−1.33, 1.05]	−.01	<.01	0.72 [−0.47, 1.90]	.07	<.01	−0.99 [−2.36, 0.38]	−.08	.01	0.29 [−0.89, 1.46]	.03	<.01
	Identifies as religious	−2.24 [−4.72, 0.23]	−.09	.01	−1.28 [−3.91, 1.35]	−.06	<.01	−2.62 [−5.23, 0.00]	−.11	.01*	−2.40 [−5.43, 0.62]	−.09	.01	−4.09 [−6.71, −1.48]	−.16	.02^‡^
	Extraversion	2.08 [0.27, 3.89]	.14	.01*	0.68 [−1.26, 2.62]	.05	<.01	0.93 [−0.99, 2.84]	.07	<.01	0.49 [−1.73, 2.71]	.03	<.01	1.37 [−0.56, 3.29]	.09	<.01
	Neuroticism	0.02 [−1.73, 1.77]	<.01	<.01	1.37 [−0.48, 3.21]	.10	.01	0.34 [−1.50, 2.18]	.02	<.01	1.27 [−0.86, 3.39]	.08	<.01	0.69 [−1.13, 2.51]	.04	<.01
	Agreeableness	−1.34 [−3.82, 1.14]	−.06	<.01	2.45 [−0.18, 5.09]	.13	.01	−1.14 [−3.76, 1.48]	−.06	<.01	0.93 [−2.06, 3.92]	.04	<.01	−0.28 [−2.88, 2.32]	−.01	<.01
	Conscientiousness	−1.14 [−3.90, 1.62]	−.05	<.01	0.21 [−2.77, 3.20]	.01	<.01	−0.06 [−2.99, 2.87]	<.01	<.01	2.25 [−1.18, 5.67]	.08	.01	−1.88 [−4.85, 1.09]	−.07	<.01
	Openness	4.38 [2.11, 6.65]	.20	.03^†^	0.50 [−1.92, 2.91]	.03	<.01	1.31 [−1.08, 3.69]	.06	<.01	2.97 [0.20, 5.74]	.13	.01*	3.22 [0.84, 5.59]	.14	.02^‡^
	Machiavellianism	0.09 [−2.10, 2.27]	<.01	<.01	0.32 [−1.99, 2.63]	.02	<.01	−1.06 [−3.35, 1.24]	−.06	<.01	−1.24 [−3.89, 1.40]	−.06	<.01	−1.80 [−4.08, 0.49]	−.09	.01
	Narcissism	−5.93 [−8.94, −2.92]	−.25	.03^†^	−1.04 [−4.23, 2.15]	−.05	<.01	−0.62 [−3.79, 2.54]	−.03	<.01	−2.02 [−5.67, 1.62]	−.08	<.01	−3.08 [−6.22, 0.05]	−.13	.01
	Psychopathy	1.87 [−1.05, 4.79]	.08	<.01	0.72 [−2.45, 3.89]	.03	<.01	−1.21 [−4.33, 1.91]	−.05	<.01	1.07 [−2.51, 4.66]	.04	<.01	−1.33 [−4.45, 1.79]	−.05	<.01
	SM	0.01 [−0.89, 0.91]	<.01	<.01	−0.79 [−1.75, 0.17]	−.10	.01	0.21 [−0.76, 1.17]	.03	<.01	0.98 [−0.13, 2.09]	.11	.01	0.67 [−0.29, 1.63]	.08	<.01
	SMxExtraversion	0.55 [−0.57, 1.67]	.06	<.01	−0.03 [−1.22, 1.15]	<.01	<.01	0.38 [−0.81, 1.57]	.04	<.01	1.10 [−0.27, 2.47]	.12	.01	0.74 [−0.45, 1.92]	.08	<.01
	SMxNeuroticism	0.23 [−0.98, 1.44]	.02	<.01	0.74 [−0.54, 2.02]	.08	<.01	1.18 [−0.09, 2.46]	.13	.01	0.93 [−0.57, 2.44]	.09	<.01	0.57 [−0.72, 1.86]	.06	<.01
	SMxAgreeableness	−0.15 [−1.82, 1.52]	−.01	<.01	0.36 [−1.42, 2.13]	.03	<.01	1.11 [−0.65, 2.88]	.09	<.01	0.92 [−1.09, 2.93]	.07	<.01	−0.37 [−2.12, 1.37]	−.03	<.01
	SMxConscientiousness	−0.12 [−2.02, 1.78]	−.01	<.01	−1.99 [−4.09, 0.12]	−.13	.01	−0.86 [−2.95, 1.22]	−.05	<.01	−1.87 [−4.40, 0.65]	−.10	.01	0.26 [−1.90, 2.42]	.01	<.01
	SMxOpenness	−1.08 [−2.54, 0.38]	−.07	.01	−0.32 [−1.87, 1.23]	−.02	<.01	−0.87 [−2.41, 0.67]	−.06	<.01	−0.23 [−2.02, 1.57]	−.01	<.01	−0.02 [−1.57, 1.52]	<.01	<.01
	SMxMachiavellianism	0.76 [−0.71, 2.22]	.06	<.01	−0.13 [−1.68, 1.42]	−.01	<.01	−0.86 [−2.40, 0.69]	−.07	<.01	−1.10 [−2.88, 0.69]	−.08	<.01	0.66 [−0.88, 2.19]	.05	<.01
	SMxNarcissism	0.41 [−1.51, 2.33]	.03	<.01	1.73 [−0.30, 3.76]	.12	.01	0.79 [−1.23, 2.81]	.05	<.01	0.13 [−2.19, 2.45]	.01	<.01	−1.57 [−3.56, 0.42]	−.10	.01
	SMxPsychopathy	−0.02 [−1.94, 1.90]	<.01	<.01	0.03 [−2.02, 2.07]	<.01	<.01	1.88 [−0.15, 3.91]	.13	.01	1.25 [−1.09, 3.59]	.08	<.01	1.87 [−0.15, 3.89]	.12	.01

SM = Time spent on social media.

*< .05.

‡< .01.

†< .001.

Step 2 showed that the addition of personality variables significantly improved the predictive ability of only the models for climate change and progressive opinions, partially supporting Hypothesis 2a. Several personality traits significantly predicted opinion strength for climate change (extraversion *sr*^2^ = .014, openness *sr*^2^ = .033, and narcissism *sr*^2^ = .037) and progressive opinions (openness *sr*^2^ = .016). All other personality traits across all issues were non-significant at step 2 of the analyses.

This hypothesis was exploratory, to determine whether there were particular traits that lend themselves to having stronger opinions, regardless of direction, or whether there were particular issues that encouraged stronger opinions in people with particular levels of each trait. Only strength of opinion on climate change and progressive opinions were significantly predicted by any personality traits. In terms of climate change, the same traits as Hypothesis 1a – extraversion, openness and narcissism – predicted significant variance. Although extraversion and openness predicted similar levels of variance in both opinion direction and opinion strength (extraversion ∆*sr*^2^ = .003, openness ∆*sr*^2^ = .006), narcissism predicted considerably more variance in opinion strength than opinion direction (∆*sr*^2^ = .013). The similarities in variance predicted by extraversion and openness across both direction and strength may suggest that most participants high in those traits expressed views ranging from neutral to positive, rather than negative, which would explain similar results in a comparison between opinion direction and opinion strength.

The considerably larger percentage of variance in opinion strength rather than direction explained by narcissism indicates that those high in narcissism shared a mix of positive and negative opinions on climate change, and by removing direction from the model, and only considering strength, that relationship was illuminated. These findings may be explained by a greater level of certainty in the opinions held by people high in narcissism. There is some research to support the idea that people high in narcissism overestimate their own abilities (Christopher et al., 2023), which may translate into being more certain they are correct, and thus having stronger opinions – independent of the direction of that opinion.

When considering progressive opinions, only openness significantly predicted any variance in opinion strength, predicting approximately the same amount of variance as in Hypothesis 1a. Extraversion, however, no longer predicted a significant amount of variance, suggesting that people high in extraversion are no more or less likely to form strong opinions on progressive issues as a whole than people that are low in extraversion. However, as the concept of opinion strength (independent of direction) does not appear to have been researched to date, further research is needed to support this conclusion.

Hypothesis 2b was partially supported, with time spent on social media significantly improving the predictive ability of the model for gender equality at step 3 (∆*R*^2^ = .015), but not for any other topic. Hypothesis 2c, exploring whether time spent on social media would moderate the relationship between personality and opinion strength on each issue, was also not supported. There was no significant improvement at step 4 of any of the models, suggesting there is no moderating effect of time spent on social media on the relationship between opinion strength and personality. These mostly non-significant results are reassuring, as it suggests that although some relationships between personality traits and overall opinion strength (regardless of direction) exist, it is unlikely that spending more time on social media is going to increase that opinion strength in general, and that any relationships that exist between personality trait and opinion strength will not be influenced by time spent on social media.

Another possibility is that the effect of social media use on opinion strength depends on which issues are more present on social media at the time of data collection. Although there is no data to support this in the current study, it is likely that the existence of a significant relationship depends on how much exposure individuals have to particular issues on social media sites. Repeated exposure to similar information can result in stronger overall opinions (Dylko et al., 2018), suggesting that were this research repeated at a time when a particular social issue was more frequently presented on social media, time spent on social media could influence the relationship between personality traits and opinion strength on that specific issue.

## Limitations and conclusion

This study is not without limitations. Although the demographic profile of the sample was broader than typical first-year university students (see Table 3 for detailed demographic features of the sample), the participants in this study were all enrolled in an undergraduate psychology degree, indicating some similarities within the sample not held by the general population. Due to the wide range of issues covered in the broader study of which this research is part, short scales were developed to measure opinions on each issue, in an effort to prevent response fatigue. These scales attained internal consistencies that ranged from acceptable to excellent, and each item had face validity; however, they were still different to the scales used in prior research, which may have contributed to the inconsistent results. For example, Annalakshmi et al. (2020) utilised the Climate Change Attitude Survey, a 15-item Likert scale measuring both intentions and beliefs about climate change, while Hodson et al. (2009) utilised a modified version of the Modern Racism Scale, a 7-item Likert scale measuring racism. Each of these scales are longer than the scales used in this research, suggesting this study may have captured less detail for opinions on each issue. Although this was inevitable, future research could focus on one or two social issues and use more detailed and extensive measures to capture opinions on those issues, which would provide easier comparison with past research. Finally, the use of the Short Dark Triad, which captures each of the Dark Triad personality traits unidimensionally, reduces the predictive abilities of the study. This scale was also chosen to reduce response fatigue, but future research could address this by shortening other elements of the survey and including multidimensional measures of each of the Dark Triad traits, in order to examine any effects of personality at a facet level.

This study found partial support for each hypothesis, but even the non-significant findings provide insight into the relationship between opinions, personality, and social media use. In terms of Hypotheses 1a and 1b, measuring how well personality traits predict direction of opinion across several social issues, and the role time spent on social media plays in that relationship, some of the hypothesised relationships were found, but many were not. While the differences between the current study and past research may be related to methodological differences (e.g., different scales used to measure opinion and/or personality), it is also possible these differences demonstrate a difference between an Australian sample and samples from Europe, Asia, and North America. Past research shows opinions on both climate change (Skamp et al., 2021) and immigration (e.g., Ueffing et al., 2015) can vary by country.

Another potential explanation for these mixed results is the measurement of time spent on social media. The highest level of use a participant could record in this study was “greater than 4 hours per day”, which may have meant participants who used social media for considerably longer than 4 hours each day were not adequately accounted for. Recent research has also found that people are inaccurate when estimating level of social media use in self-report measures (Steele et al., 2023), which may also have impacted these results. Finally, the inconsistencies with past research may also relate to the HMRA used in this study, adding precision by accounting for many key demographic variables prior to drawing conclusions about the hypotheses. This step has not always been taken in past research.

Hypotheses 2a and 2b were exploratory by nature, investigating whether opinion strength – regardless of direction – related to levels of particular personality traits, and the role time spent on social media plays in that relationship. Although the results were largely non-significant, they are still encouraging. They suggest that being higher in particular personality traits does not generally encourage stronger opinions when direction of opinion is not considered. Although polarisation is still a concern when considering the direction of an opinion, people high in particular personality traits do not seem to polarise for the sake of having stronger opinions. The content of the issue appears to matter most. The results relating to time spent on social media across both Hypotheses 1b and 2b may also be explained by evaluating which issues were most prevalent on social media platforms at the time of data collection, and controlling for this in future research. It is likely that as events unfold in society related to particular issues, individuals are exposed to those issues more frequently on social media, leading to a higher probability that time spent on social media will relate to the opinions they form on those issues.

This research has provided another perspective to the relationships between personality traits and opinions, with some results differing from previous research, suggesting potential differences between an Australian population and other populations. However, as the sample was not representative of the general Australian population, the veracity of these differences requires further investigation. The findings of this study suggest that social media use does not generally play a considerable role in the relationship between personality and opinions, even though it is often found to be related to both variables independently. As such, although strong opinions will still be encouraged independently by social media use and personality traits, there is no evidence to suggest that individuals high in certain personality traits will become more polarised on social media than those with lower levels of those traits.

## Data Availability

The data that support the findings of this study are available from the corresponding author, MC, upon reasonable request. Australian Government [Research Training Program Scholarship].

## Appendix A

Questions
***Asked to Measure Opinions on Each Issue***

### Climate Change

Q1. I believe climate change is a serious issue

Q2. I believe Australia should spend more money on climate research

Q3. I agree with further taxes on big business and/or corporations that emit carbon emissions in excessive amounts

Q4. I believe the government is doing enough to combat climate change (R)

### Religious Freedoms

Q1. I believe public figures should be allowed to express religious views publicly without personal or professional repercussion (R)

Q2. I believe religious organisations and businesses should be allowed to refuse employment based on religious beliefs (R)

Q3. I believe religious organisations and businesses should be allowed to refuse service based on religious beliefs (R)

### Immigration

Q1. All boats carrying asylum seekers should be turned back (R)

Q2. Australia should permit more immigrants than it already does

Q3. Immigrants from non-English speaking countries should only be admitted if they are proficient in English (R)

Q4. All immigrants can retain their cultural values without being any less Australian.

### Gender Equality

Q1. The gender pay gap is a serious problem

Q2. School-aged girls should be more encouraged to study STEM (Science, Technology, Engineering and Maths) subjects

Q3. Toys in retail shops should not be sorted by gender
